# Changes in circulating microRNAs after radiochemotherapy in head and neck cancer patients

**DOI:** 10.1186/1748-717X-8-296

**Published:** 2013-12-28

**Authors:** Isolde Summerer, Maximilian Niyazi, Kristian Unger, Adriana Pitea, Verena Zangen, Julia Hess, Michael J Atkinson, Claus Belka, Simone Moertl, Horst Zitzelsberger

**Affiliations:** 1 Research Unit Radiation Cytogenetics, Helmholtz Center Munich, Ingolstaedter Landstr 1, 85764, Neuherberg, Germany; 2 Department of Radiation Oncology, University of Munich, Marchioninistr 15, 81377, Munich, Germany; 3 Institute of Radiation Biology, Helmholtz Center Munich, Ingolstaedter Landstr 1, 85764, Neuherberg, Germany; 4 Clinical Cooperation Group ‘Personalized Radiotherapy of Head and Neck Cancer’, Helmholtz Center Munich, Ingolstaedter Landstr 1, 85764, Neuherberg, Germany

**Keywords:** Head and neck cancer, Circulating non-coding RNA, Biomarker, Radiotherapy outcome, HNSCC cell culture model

## Abstract

**Introduction:**

Circulating microRNAs (miRNAs) are easily accessible and have already proven to be useful as prognostic markers in cancer patients. However, their origin and function in the circulation is still under discussion. In the present study we analyzed changes in the miRNAs in blood plasma of head and neck squamous cell carcinoma (HNSCC) patients in response to radiochemotherapy and compared them to the changes in a cell culture model of primary HNSCC cells undergoing simulated anti-cancer therapy.

**Materials and methods:**

MiRNA-profiles were analyzed by qRT-PCR arrays in paired blood plasma samples of HNSCC patients before therapy and after two days of treatment. Candidate miRNAs were validated by single qRT-PCR assays. An *in vitro* radiochemotherapy model using primary HNSCC cell cultures was established to test the possible tumor origin of the circulating miRNAs. Microarray analysis was performed on primary HNSCC cell cultures followed by validation of deregulated miRNAs via qRT-PCR.

**Results:**

Unsupervised clustering of the expression profiles using the six most regulated miRNAs (*miR-425-5p*, *miR-21-5p*, *miR-106b-5p*, *miR-590-5p*, *miR-574-3p*, *miR-885-3p*) significantly (p = 0.012) separated plasma samples collected prior to treatment from plasma samples collected after two days of radiochemotherapy. MiRNA profiling of primary HNSCC cell cultures treated *in vitro* with radiochemotherapy revealed differentially expressed miRNAs that were also observed to be therapy-responsive in blood plasma of the patients (*miR-425-5p*, *miR-21-5p*, *miR-106b-5p*, *miR-93-5p*) and are therefore likely to stem from the tumor. Of these candidate marker miRNAs we were able to validate by qRT-PCR a deregulation of eight plasma miRNAs as well as *miR-425-5p* and *miR-93-5p* in primary HNSCC cultures after radiochemotherapy.

**Conclusion:**

Changes in the abundance of circulating miRNAs during radiochemotherapy reflect the therapy response of primary HNSCC cells after an *in vitro* treatment. Therefore, the responsive miRNAs (*miR-425-5p*, *miR-93-5p*) may represent novel biomarkers for therapy monitoring. The prognostic value of this exciting observation requires confirmation using an independent patient cohort that includes clinical follow-up data.

## Introduction

Surgical treatment of head and neck squamous cell carcinoma (HNSCC) is limited by the complex anatomy of the tumors and the associated risk of morbidity. Hence, for many patients an alternative treatment strategy of definitive radiotherapy alone or in conjunction with chemotherapy or immunotherapy is required [1]. Despite considerable progress in treatment options, disease recurrence and metastasis with a very strong impact on long-term survival are still the major challenges [2].

MicroRNAs (miRNAs) are evolutionarily conserved small RNAs, representing a class of regulators of posttranscriptional gene expression. There are more than 2000 mature miRNAs currently annotated in the human genome miRBase (release 19.0) [3] potentially targeting over 60% of all proteins [4]. Fundamental cellular processes including development, apoptosis [5], cell cycle control, proliferation [6] and DNA-damage repair [7] are influenced by miRNAs. During the last decade alterations in miRNA expression have been associated with a number of human diseases, including cancer (reviewed in [8]). The recent discovery of miRNAs in body fluids such as cerebrospinal fluid [9] and blood plasma [10] opens up the possibility of using miRNAs as minimally invasive biomarkers for the prediction of clinical endpoints such as overall survival [11,12]. The well described ‘onco-miR’ *miR-21* has already been identified as a valuable plasma biomarker with high prognostic power in esophageal squamous cell carcinoma [13] and gastric cancer [14].

Since the expression of miRNAs is known to be altered by ionizing radiation at both the cellular level [15] and plasma levels of mice [16], they might serve as easily accessible predictors of the individual response to radiation therapy. Additionally, miRNAs regulate drug sensitivity [17] and influence radioresistance [18-20]. This offers the possibility of using specific plasma miRNAs as biomarkers for optimized treatment decisions.

The present study aimed to identify blood plasma miRNAs showing a response to radiochemotherapy and further to clarify the origin of these miRNAs in order to use them as minimally invasive tools for therapy monitoring.

For this purpose we compared miRNA levels in samples of blood plasma from HNSCC patients prior to treatment and after the completion of the first two fractions of therapeutic irradiation. MiRNAs displaying altered concentration levels after treatment were further analyzed in peripheral blood mononuclear cells (PBMC) of the same patients to test the hypothesis of PBMC-derived alterations of the plasma miRNAs. Moreover, we established an *in vitro* radiochemotherapy model using primary HNSCC cells in order to investigate a potential tumor origin of the therapy-responsive miRNAs in blood plasma.

## Materials and methods

### Patient samples

Plasma miRNA analysis was performed on 18 patients (17 HNSCC, 1 esophageal adenocarcinoma) treated with local X-ray-irradiation using a linear accelerator (6 MV, Siemens Mevatron M or ELEKTA Synergy®). After a planning (PET-) CT scan 70 Gy were applied to the macroscopic tumor and involved lymph nodes in daily dose fractions of 2 Gy five days per week. The adjuvant lymphatics were irradiated with up to 50 Gy and the high-risk lymphatics (adjacent to the involved lymph node levels) with up to 60 Gy.

16 out of 18 patients received concurrent chemotherapy (12 patients received 5-fluorouracil (5-FU) plus mitomycin C (MMC) [21], 3 patients MMC and 1 patient cisplatin weekly). None of the patients underwent surgical treatment. 5-FU treatment was usually applied on each of the first 5 days of therapeutic irradiation whereas MMC was applied only on day 5 and day 36 during radiotherapy. Patient characteristics are listed in Table 1.

**Table 1:** Patient characteristics

**Characteristic**		**Number of patients**
*Gender*		
	male	14
	female	4
*Median age, years*		57.9
*Age range, years*		45.1–80.6
*Tumor site*		
	Larynx	5
	Oropharynx	3
	Mouth floor	2
	Tongue	2
	Esophagus	1
	Hypopharynx	1
	Maxilla	1
	Nasopharynx	1
	Sinuses	1
	Soft palate	1
*T-Stage*		
	I	4
	II	2
	III	6
	IV	6
*N-Stage*		
	N0	4
	N1	4
	N2	10
*M-Stage*		
	M0	16
	M1	2
*Concomitant therapy (in addition to radiotherapy)*		
	5-FU + MMC	12 (patient 3, 5–7, 9–13, 15–17)
	MMC	3 (patient 1, 14, 18)
	Cisplatin	1 (patient 8)
	Cetuximab	1 (patient 4)
	none	1 (patient 2)
*Acute Toxicity*		
	severe	11
	moderate	5
	n.a.	2

5-FU = 5-fluorouracil; MMC = mitomycin C;

n.a. = not available.

After obtaining ethical approval and informed consent, 15 ml of EDTA-peripheral blood were collected from each patient prior to the first fraction of therapy and within one hour after the second fraction of therapeutic irradiation. EDTA-blood samples were centrifuged at 350 × g for 10 min within two hours after collection to obtain plasma. To avoid cellular contamination the plasma samples were re-centrifuged at 1,200 × g for 3 min and subsequently at 14,000 × g for 10 min to remove cell debris.

PBMC were isolated using Ficoll gradient centrifugation.

Samples were stored at -20°C until further analysis or at -80°C for long term storage.

The study was approved by the ethics committee of the University of Munich (Germany).

### Primary HNSCC cells

Primary tumor cells were obtained from the fresh tumor biopsies of two HNSCC cases, HN1957 (left maxilla/left nasal floor) and HN2092 (right floor of mouth) received from the Wales Cancer Bank, UK [22]. The tumor tissues were rinsed with PBS, minced and cultivated in keratinocyte media supplemented with L-glutamine, penicillin/streptomycin, EGF, BPE, CaCl_2_, F-12, DMEM (all components: Life Technologies) and 10% FBS at 37°C and 5% CO_2._ To ensure the exclusive cultivation of epithelial cells from tissue particles, fibroblast cells were identified via frequent microscopy and removed by scraping. The FBS-content of the media was continuously reduced down to serum-free cultivation. The epithelial origin of both cultures was assessed by positive cytokeratin staining via immunohistochemistry (Additional file 1). Characteristics of the two primary HNSCC cultures are listed in Table 2.

**Table 2:** Characteristics of primary HNSCC cell cultures

**Characteristic**	**HN1957**	**HN2092**
*Gender of patient*	female	male
*Age at diagnosis, years*	85	73
*Tumor site*	left maxilla/left nasal floor	right floor of mouth
*TNM*	n.a.	pT4pN0
*Cell type*	epithelial	epithelial

n.a. = not available.

### Treatment of HNSCC cells

**Figure 1:**
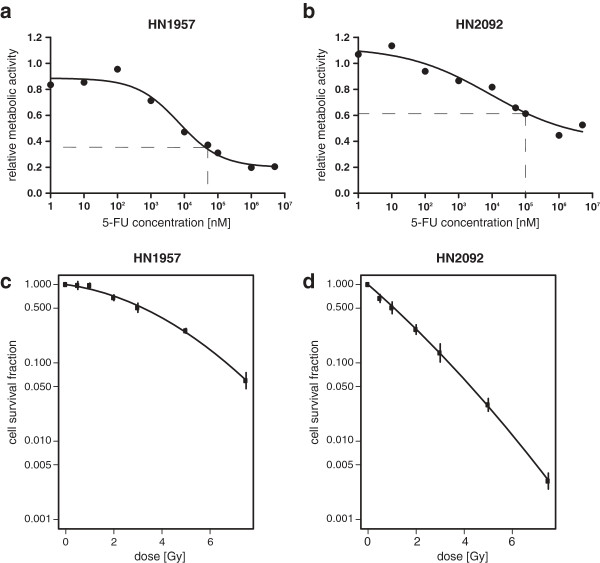
**Sensitivity of primary HNSCC cell cultures to 5-FU treatment and ionizing radiation.** Cell viability (XTT-assay) of HN1957 **(a)** and HN2092 **(b)** 48 h after 5-FU treatment relative to DMSO-controls. Each data point represents the mean of three biological replicates. Dashed lines mark the 5-FU concentrations selected for *in vitro* radiochemotherapy modeling. Survival curves were generated by colony forming assay for HN1957 **(c)** 6 days after irradiation and HN2092 **(d)** 12 days after irradiation. Error bars represent the standard deviation of three biological replicates.

To model as closely as possible the most frequent chemotherapy treatment we tested different 5-FU treatment options using the primary HNSCC cultures. Cell viability was measured using the XTT cell proliferation kit II (Roche) for both cell cultures after applying a wide range of 5-FU doses (Figure 1a, b). The 5-FU concentrations were selected to be relatively high, however lying within the linear region of the dose–response curve. Thus, HN1957 cells were treated with 50 μM 5-FU and HN2092 cells, as they showed less sensitivity towards the agent, were treated with 100 μM 5-FU.

Cells were seeded in 60 mm-dishes and 24-well plates for RNA assays and cell viability assays, respectively. On the following day, cells were irradiated with 2 Gy using a ^137^Cs source and treated with 5-FU (Sigma; solved in DMSO). Controls were treated with the corresponding volumes of DMSO and sham-irradiated. 24 h after the first irradiation a second fraction of 2 Gy was applied to the 5-FU-treated cells followed by incubation for 1 h at 37°C. Cells were harvested by trypsinization and stored at -20°C until further processing.

### Cell viability assay (XTT)

In order to determine cell viability of cells treated with *in vitro* radiochemotherapy or 5-FU treatment alone, 24-well plates were used for three biological and two technical replicates of each condition. XTT-assay was conducted using 350 μl of Medium and 150 μl of XTT-labeling mixture. Measurement was performed 24 h after 5-FU treatment.

### Colony forming assay

In order to determine the radiation sensitivity of the primary HNSCC cells, the colony forming assay was performed in 6-well plates (Figure 1c, d). To ensure stable growth of the primary cells, a feeder layer consisting of the same HNSCC cells was used. Feeder cells were irradiated with 50 Gy in a ^60^Co source before seeding in the 6-well plates (10,000 cells/well). The following day, fresh cells for colony formation were plated on the feeder layer. On the following day the cells were irradiated using a ^137^Cs source with 0, 0.5, 1, 2, 3, 5 or 7.5 Gy. HN1957 colonies were stained with Giemsa six days and HN2092 colonies 12 days after irradiation. Colonies consisting of at least 50 cells were scored. Each colony formation assay was carried out in triplicate and repeated three times.

### RNA extraction

Total RNA was extracted from 300 μl plasma using the mirVana miRNA Isolation Kit (Ambion) according to the manufacturer’s protocol with the following modification: 1,000 μl of lysis buffer was added to 300 μl of plasma. 1.04 fmol of synthetic *cel-miR-39 (C. elegans)* was added to each plasma sample (300 μl) after the protein denaturation step to normalize sample-to-sample variation in RNA recovery. RNA was finally eluted into 50 μl of pre-heated (95°C) nuclease-free water.

RNA from PBMC was extracted with the mirVana miRNA Isolation Kit (Ambion) according to the manufacturer’s protocol. RNA purity was measured by spectrophotometry (OD 260/280 ratio) using a Nanodrop ND-1000 (Thermo Scientific). Ratios were in the range of 1.89 to 2.09.

Extraction of total RNA from primary HNSCC cells was performed using the miRNeasy mini kit (Qiagen) according to the manufacturer’s protocol without DNase digest or small RNA enrichment. OD 260/280 ratios, measured on a Nanodrop ND-1000 (Thermo Scientific), were in the range of 1.92 to 2.04. Additionally, RNA quality was assessed prior to the Agilent microarray experiments using an Agilent 2100 Bioanalyzer (Agilent Technologies). The computed RNA integrity numbers (RINs) ranged from 9.3 to 10.0.

### MiRNA profiling in patient plasma

TaqMan Array Human MicroRNA A Cards v2.0 (Applied Biosystems) representing 377 mature human miRNAs were used to obtain miRNA profiles of the blood plasma samples. Reverse transcription was performed using the TaqMan miRNA reverse transcription kit (Applied Biosystems) in combination with the stem-loop megaplex primer pool set A v2.1. Because of the low abundance of circulating miRNA in the starting material we used a fourfold volume of the reaction mix, prepared according to the manufacturer’s protocol adding 12 μl of total plasma RNA.

For the subsequent quantitative real-time PCR 30 μl of cDNA was used for each array and PCR was carried out on an Applied Biosystems 7900HT. The reaction mixtures were incubated at 50°C for 2 min and 95°C for 10 min, followed by 45 cycles of 95°C for 30 s and 60°C for 1 min. All Ct values were normalized using the median Ct value of all detectable miRNAs on the array.

### MicroRNA profiling in HNSCC cells

To analyze the effects of *in vitro* radiochemotherapy on the cellular miRNA expression levels Sure Print G3 human 8x60k miRNA microarrays (Agilent Technologies) were used covering 1205 human miRNAs (Sanger miRBase release 16). 100 ng of total RNA was dephosphorylated and labeled with cyanine 3-cytidine biphosphate including a labeling spike-in solution (Agilent Technologies) to assess the labeling efficiency. After purification of the labeled RNA, the samples were hybridized on the arrays including a hybridization spike-in solution (Agilent Technologies) to monitor hybridization efficiency. Arrays were scanned with a G2505C Sure Scan Microarray Scanner (Agilent Technologies) using Scan Control software. For data processing the following software was used: Feature extraction 10.7 (Agilent Technologies) and GeneSpring 11.5. MiRNA analysis was conducted with three biological and two technical replicates for each data point. All steps were performed according to the manufacturer’s protocol. The raw data were imported into the R statistical platform [23]. The total microRNA gene signal (TGS) was computed by the Agilent Extraction feature. The resulting signal was normalized to the mean signal of all arrays for compensation of systematic technical slide-to-slide variations. A differential expression analysis was performed using the linear model components implemented in *limma*[24] and empirical Bayes methods were applied in order to attain moderated statistics [25].

### Real-time PCR quantification of individual miRNAs

Reverse transcription was performed using the TaqMan miRNA reverse transcription kit and miRNA-specific stem-loop primers (Applied Biosystems). Reverse transcription was performed on a Cyclone PCR system (Peqlab) according to the manufacturer’s protocol. For miRNA assays in plasma samples 3 μl of cDNA was used for reverse transcription because of the low RNA content. Real-time PCR was performed in duplicates and included non-template negative controls. PCR was performed on a ViiA 7 real-time PCR System (Applied Biosystems) following the manufacturer’s protocol. For plasma samples the miRNA concentration levels were normalized to the spiked-in *cel-miR-39*, for cellular samples the U6 snRNA was used for normalization. Fold changes were calculated using the 2^-ΔΔCt^ method [26].

### Statistical analysis

To identify differentially expressed miRNAs in plasma samples of patients prior to radiotherapy and after the second fraction of therapeutic irradiation Wilcoxon test was performed. All miRNAs that were detected in less than 30% of either control samples or irradiated samples were excluded from further analysis. Unsupervised hierarchical clustering was performed using the top distinctive miRNAs applying the parameters maximum distance and Ward’s method. Fisher’s exact test was applied to check for significant clustering of samples from patients prior to treatment and those collected after treatment. *P* values < 0.05 were considered statistically significant.

Correlation coefficients for miRNA changes in PBMC and plasma as well as in arrays and single assays were calculated using Pearson correlation. All statistical analyses were performed using The R Project for Statistical Computing [23].

## Results

**Figure 2:**
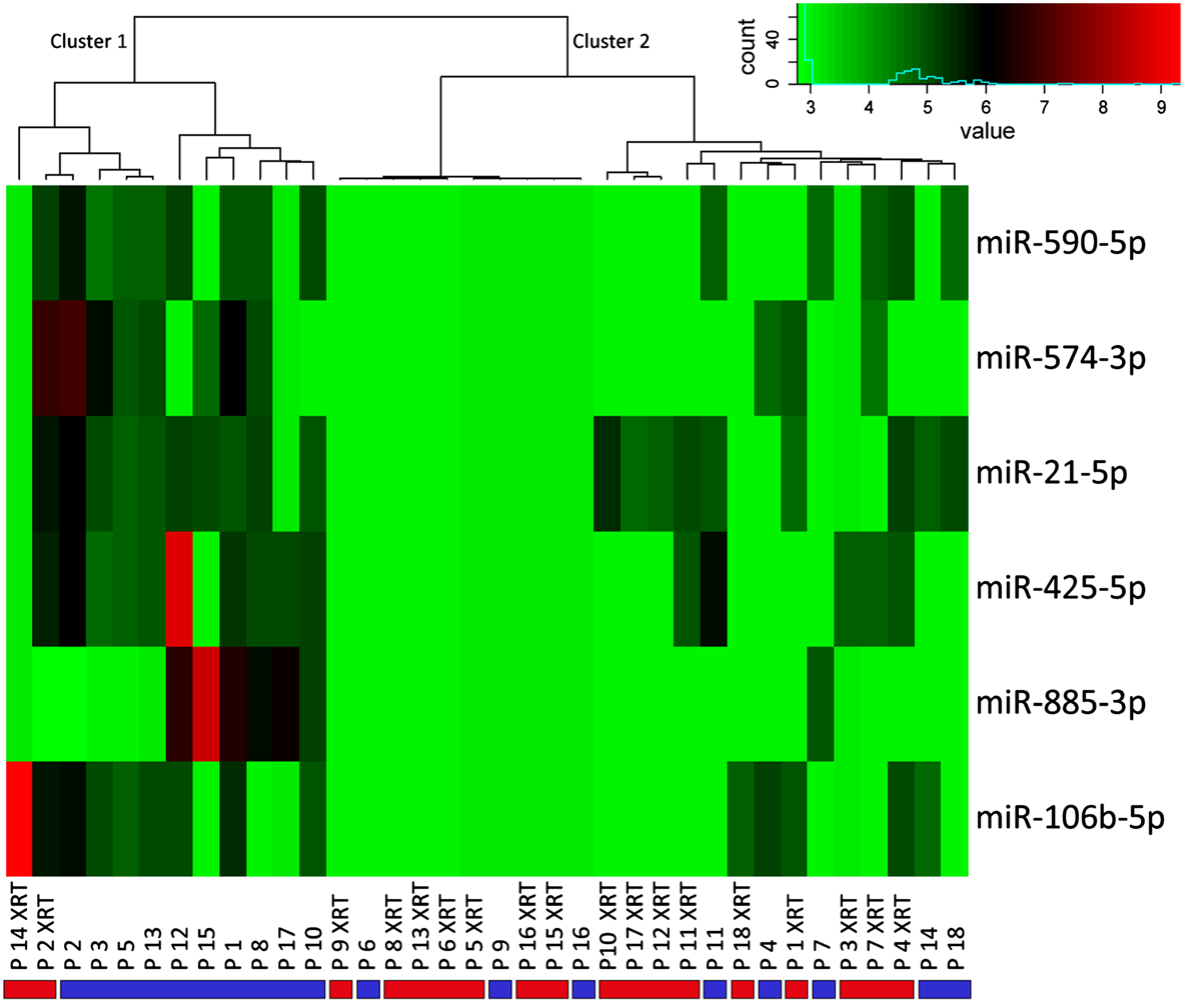
**Unsupervised hierarchical cluster analysis of samples collected before therapy and after two days of treatment.** Samples collected prior to treatment are marked with blue bars, samples collected after two days of treatment (XRT = radiotherapy) are marked with red bars. *Cluster 1* and *Cluster 2* indicate the two main clusters emerging from unsupervised cluster analysis using blood plasma concentration levels of the top six distinctive miRNAs.

MiRNA profiling of the plasma samples from 18 head and neck cancer patients revealed 54 miRNAs with altered expression levels after treatment (Additional file 2). Unsupervised hierarchical clustering using the expression levels of the top six significantly deregulated miRNAs revealed two main clusters. Cluster 1 represents ten samples collected prior to treatment and two samples collected after treatment, whereas cluster 2 consists of eight pre-treatment samples and 16 post-treatment samples. Consequently, the miRNA profiles of these six miRNAs differentiated significantly (p = 0.012) between samples collected before therapy and those collected after two days of treatment (Figure 2). For technical validation of the array data eight miRNAs (*miR-590-5p, miR-574-3p, miR-425-5p, miR-885-3p, miR-21-5p, miR-28-3p, miR-195-5p, miR-191-5p*) were selected from the top list of deregulated miRNAs. Additionally, *miR-150-5p* and *miR-142-3p*, both known to play a role in esophageal carcinoma [27,28], were used for validation. For these ten miRNAs the differences between the two groups were validated using single qRT-PCR assays. The correlation coefficients of the normalized Ct values demonstrated a good correlation between the results of the TaqMan low density array and the TaqMan single assays for most of the tested miRNAs (Additional file 3). The single assays of *miR-590-5p* and *miR-885-3p* were below the detection level in almost all plasma samples and were consequently excluded from further analyses.

Many plasma miRNAs are known to originate from peripheral blood mononuclear cells (PBMC) [29]. Therefore, we measured the expression levels of the candidate therapy biomarker miRNAs (*miR-574-3p, miR-425-5p, miR-21-5p, miR-28-3p, miR-195-5p, miR-191-5p, miR-150-5p, miR-142-3p*) in PBMC samples obtained from the same patients. Correlation coefficients of the normalized Ct values indicated that the changed miRNA levels after treatment do not originate from PBMC (Additional file 4).

In order to further investigate whether the plasma miRNA changes following tumor therapy originate from the tumor cells we established an *in vitro* radiochemotherapy model using primary HNSCC from two different patients, HN1957 and HN2092 (Table 2).

**Figure 3:**
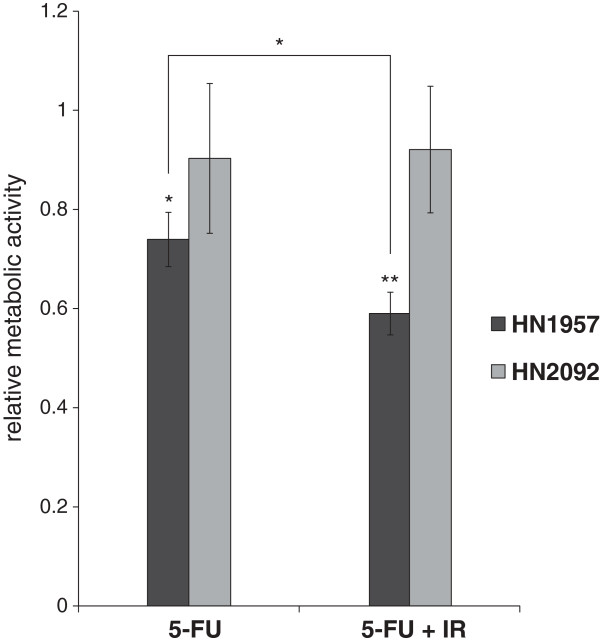
**Cell viability (XTT-assay) of primary HNSCC cell cultures.** Absorption was measured 24 h after exclusive 5-FU treatment and 5-FU treatment in combination with 2 x 2 Gy *in vitro* irradiation relative to DMSO-controls. Error bars represent the standard deviation of three biological replicates (*p < 0.05, **p < 0.01).

For assessment of the sensitivity of the primary tumor cells to 5-FU and to ionizing radiation dose-response curves (Figure 1a, b) and survival curves (Figure 1c, d) were generated, respectively. HN2092 cells showed higher sensitivity to ionizing radiation but lower sensitivity to 5-FU treatment compared to HN1957 cells. In order to simulate the treatment of the patient samples in the present study cells were irradiated with 2 Gy followed by treatment with 5-FU and on the following day irradiation with a second 2 Gy fraction. Subsequent measurement of the cell viability (24 h after 5-FU treatment) showed no significant effect of 5-FU or irradiation on HN2092 cell cultures, whereas cell viability of HN1957 cell cultures was already reduced by 5-FU treatment alone with a significant additive effect of irradiation on the cell viability (Figure 3).

MiRNA profiling using Agilent microarrays in the sham-irradiated and DMSO-treated control cells compared to cells treated with *in vitro* radiochemotherapy revealed several miRNAs that differentiate control cells and treated cells for HN1957 and HN2092 (Additional files 5 and 6). We selected three miRNAs (*miR-425-5p, miR-21-5p, miR-106b-5p*) that were altered after *in vitro* treatment and were also in the top list of therapy-responsive miRNAs in patient plasma samples after *in vivo* tumor therapy. Additionally, *miR-93-5p* was included as it shows deregulation after *in vitro* treatment and *in vivo* treatment in most of the patients, although it is not among the top significant therapy-responsive miRNAs due to the different directions of regulation (Additional file 7). For these miRNAs we conducted TaqMan single assays in control and treated cells to validate the array results. The qRT-PCR assays confirmed an up-regulation of *miR-425-5p* with a *p* value close to the significance level (p = 0.552) in HN1957 cell cultures, while for *miR-93-5p* a significant down-regulation (p < 0.001) in HN2092 cell cultures became apparent (Table 3). Thus, at least two of the four treatment-regulated plasma miRNAs are likely to be associated with the direct response of tumor cells to treatment.

**Table 3:** MicroRNAs significantly deregulated after radiochemotherapeutic treatment in primary HNSCC cell cultures and in plasma of HNSCC patients

	**Agilent microarray**		**TaqMan single qRT-PCR assay**	
**miRNA**	**HN1957**	**HN2092**	**HN1957**	**HN2092**
	**FC (*p *** **value)**	**FC (*p *** **value)**	**FC (*p *** **value)**	**FC (*p *** **value)**
*miR-425-5p*	1.27 (0.004)	-	1.28 (0.052)	-
*miR-21-5p*	1.11 (0.031)	-	1.11 (0.543)	-
*miR-106b-5p*	0.95 (0.042)	-	1.04 (0.830)	-
*miR-93-5p*	0.92 (0.023)	0.96 (0.001)	1.03 (0.552)	0.84 (0.000)

FC = fold change.

## Discussion

The present study aimed to identify circulating miRNA markers in the plasma of HNSCC patients that are indicative of the efficacy of radiochemotherapy. Our finding that a limited panel of deregulated plasma miRNAs discriminates between pre- and post-radiochemotherapy samples (Figure 2) supports previous evidence [16] for the potential use of plasma miRNAs as radiation-responsive biomarkers. Moreover, several candidates altered after the second fraction of therapeutic irradiation, such as *miR-93-5p*, *miR-142-3p*, *miR-106b-5p*, *miR-191-5p* and *miR-21-5p* (Additional file 2) have been previously described to be induced by ionizing radiation [30].

Although it is known that circulating miRNAs are present in stable forms, there is still uncertainty about their transport and cellular origin (reviewed in [31]). Since circulating miRNAs may originate from blood cells [29] we tested the hypothesis that the observed therapy-responsive plasma miRNAs originate from peripheral blood mononuclear cells (PBMC) of the same patients. Our analyses of the miRNA profiles of PBMC revealed no significant correlation between the miRNA expression in PBMC and plasma in patients before or after treatment (Additional file 4). We conclude that the plasma miRNA changes detected in response to anti-cancer therapy are most likely not originating from circulating PBMC.

It has frequently been hypothesized that plasma miRNAs originate from tumor cells, either based on active secretion [32] or in apoptotic bodies released from dead tumor cells [33]. This is supported by the present study as several candidates among the top significant plasma miRNAs that were altered after radiochemotherapy (Additional file 2) have already been reported to be tumor markers before. *MiR-195-5p, miR-574-3p* and *miR-28-3p* have been described as being differentially expressed in esophageal squamous cell carcinoma [34,35]. *MiR-191-5p* and *miR-21-5p* play a role in lung cancer diagnosis and prognosis [36], while *miR-21-5p* in plasma also is known to serve as prognostic marker in esophageal cancer patients [13]. Taken together, these reports indicate that the observed changes in plasma miRNAs in our study might be related to therapy effects on tumor cells.

To further strengthen this hypothesis we investigated the miRNA expression of primary HNSCC cell cultures under simulated radiochemotherapeutic treatment *in vitro*. The results of the miRNA expression analysis of this *in vitro* model further supported the assumption that the observed plasma miRNA changes are likely to originate from the tumor cells. We were able to show a significant deregulation of four plasma miRNAs in HN1957 cell cultures (*miR-425-5p*, *miR-21-5p*, *miR-106b-5p*, *miR-93-5p*) and one plasma miRNA in HN2092 cell cultures (*miR-93-5p*) by microarray profiling. The observation that the same miRNAs were altered in both tumor cells and plasma leads us to conclude that plasma miRNA changes following radiochemotherapeutic treatment are the result of miRNA release from damaged tumor cells. The observed differences in deregulated miRNAs between the two cell cultures, however, were not comparable. The two primary HNSCC cell cultures had different responses to radiochemotherapeutic treatment. This may be due to differences in the responsiveness of the cell cultures. Indeed, the cell viability assay (XTT) exhibited a significant effect 24 h after irradiation and 5-FU treatment on HN1957 cells, but not on HN2092 cells (Figure 3). This reflects a higher short-term toxicity of the treatment on HN1957 cell cultures compared to HN2092 cell cultures, whereas the long-term effect of irradiation is stronger on HN2092 cell cultures as shown in the colony forming assay (Figure 1c, d). The common deregulation of 43 miRNAs in both primary HNSCC cell cultures (Additional files 5 and 6) suggests a similar miRNA response to therapeutic treatment independent from the individual tumor. We also observed differences in the miRNA response of both HNSCC cell cultures, which might reflect variations in the individual sensitivity to radiation and chemotherapeutic treatment (Figure 1).

The major finding of this study, i.e. therapy-related miRNA expression in the blood plasma is similar to miRNA changes reported for treated tumor cells, is not only based on global miRNA array screens but was also technically validated by qRT-PCR studies of the most distinctive miRNAs (Table 3). We identified *miR-425-5p* (HN1957) and *miR-93-5p* (HN2092) as top candidates of commonly deregulated miRNAs in primary HNSCC cell cultures and blood plasma of HNSCC patients.

In conclusion, the present study compared miRNA data from blood plasma of radiochemotherapy-treated HNSCC patients and from an *in vitro* model of primary tumor cell radiochemotherapy. This comparison sheds light on the origin and potential use of circulating miRNAs in the blood plasma of HNSCC patients as therapy-responsive biomarkers. We will further integrate these therapy-responsive plasma miRNAs with clinical data of the patients (e.g. overall survival) and seek for independent validation in another patient cohort in order to evaluate their potential for prognostic use. The established HNSCC cell culture model further allows us to investigate the molecular interactome of the deregulated miRNA candidates by adding mRNA expression data and subsequent integrative data analysis.

The current study strongly suggests that alterations of miRNAs following radiochemotherapy in the blood plasma are associated with the tumor response to therapy and therefore might represent novel biomarkers for therapy monitoring.

## Competing interests

The authors declare that they have no competing interests.

## Authors’ contributions

IS: Experiments, manuscript, MN: Patient recruitment, ethics approval, AP: Biostatistics analysis, KU: Bioinformatics and biostatistics analysis, Support for experimental design/concept, VZ: Establishment of primary HNSCC cell cultures, JH: Support for microarray experiments, MJA: Support for planning of experiments, CB: Review of clinical data of patients, SM: Conceived the project, designed the *in vivo* study, HZ: Study design and critical revision of the manuscript. All authors read and approved the final manuscript.

## Supplementary Material

Additional file 1 Immunohistochemical cytokeratin-staining of primary HNSCC cultures. Click here for file

Additional file 2 Therapy-responsive microRNAs in plasma samples of 18 head and neck cancer patients. Click here for file

Additional file 3 Correlation coefficients of normalized Ct values (ΔCt) of plasma miRNAs analyzed with arrays and single assays. Click here for file

Additional file 4 Correlation coefficients of normalized Ct values (ΔCt) of miRNAs analyzed with TaqMan single assays in PBMC and plasma. Click here for file

Additional file 5 **Significantly deregulated microRNAs in HN1957 primary cell cultures after *in vitro *** **radiochemotherapy.** Click here for file

Additional file 6 **Significantly deregulated microRNAs in HN2092 primary cell cultures after** ***in vitro*** **radiochemotherapy.** Click here for file

Additional file 7 **Expression levels of** ***miR-93-5p*** **in plasma of 18 head and neck cancer patients prior and post treatment.** Click here for file
