# Erratum: Changes in circulating microRNAs after radiochemotherapy in head and neck cancer patients

**DOI:** 10.1186/s13014-015-0394-8

**Published:** 2015-04-24

**Authors:** Isolde Summerer, Maximilian Niyazi, Kristian Unger, Adriana Pitea, Verena Zangen, Julia Hess, Michael J Atkinson, Claus Belka, Simone Moertl, Horst Zitzelsberger

**Affiliations:** Research Unit Radiation Cytogenetics, Helmholtz Center Munich, Ingolstaedter Landstr 1, 85764 Neuherberg, Germany; Department of Radiation Oncology, University of Munich, Marchioninistr 15, 81377 Munich, Germany; Institute of Radiation Biology, Helmholtz Center Munich, Ingolstaedter Landstr 1, 85764 Neuherberg, Germany; Clinical Cooperation Group ‘Personalized Radiotherapy of Head and Neck Cancer’, Helmholtz Center Munich, Ingolstaedter Landstr 1, 85764 Neuherberg, Germany

## Erratum

After the publication of this work [1], a few points were brought to our attention:

The clinical data of patient 2 are incorrect. A revised version of Table 1 [1] showing the corrected patient characteristics is presented here. Consequently, the description of the patient cohort in the Materials and Methods section is incorrect. The correct description is: “*Plasma miRNA analysis was performed on 18 HNSCC patients treated with local X-ray-irradiation using a linear accelerator […].*

**Table 1:** **Patient characteristics**

**Characteristic**	**Number of patients**
*Gender*	
Male	14
Female	4
*Median age, years*	57.5
*Age range, years*	45.1–80.6
*Tumor site*	
Larynx	5
Oropharynx	3
Mouth floor	2
Tongue	2
Hypopharynx	1
Maxilla	1
Nasopharynx	1
Sinuses	1
Soft palate	2
*T-Stage*	
I	4
II	3
III	5
IV	6
*N-Stage*	
N0	3
N1	4
N2	11
*M-Stage*	
M0	17
M1	1
*Concomitant therapy (in addition to radiotherapy)*	
5-FU + MMC	13 (patient 2, 3, 5–7, 9–13, 15–17)
MMC	3 (patient 1, 14, 18)
Cisplatin	1 (patient 8)
Cetuximab	1 (patient 4)
*Acute toxicity*	
Severe	12
Moderate	5
n.a.	1

5-FU = 5-fluorouracil; MMC = mitomycin C; n.a. = not available.

*17 out of 18 patients received concurrent chemotherapy (13 patients received 5-fluorouracil (5-FU) plus mitomycin C (MMC) [21], 3 patients MMC and 1 patient cisplatin weekly)”.*

There is a typing error in the Results section [1] concerning the p value of the up-regulation of *miR-425-5p*. The correct sentence is:

*“The qRTPCR assays confirmed an up-regulation of miR-425-5p with a p value close to the significance level (p = 0.052) […]”.*

The corrections do not affect any results or conclusions of the published work.

## References

[1] Summerer I, Niyazi M, Unger K, Pitea A, Zangen V, Hess J (2013). Changes in circulating microRNAs after radiochemotherapy in head and neck cancer patients. Radiat Oncol.

