# Supplementary material for: Changes in circulating microRNAs after radiochemotherapy in head and neck cancer patients
Source: Radiat Oncol. 2013 Dec 28;8:296. doi: 10.1186/1748-717X-8-296 (PMC3882107; doi:10.1186/1748-717X-8-296)
Supplement: Additional file 2 — Therapy-responsive microRNAs in plasma samples of 18 head and neck cancer patients. [file 1748-717X-8-296-S2.doc]

Additional file 2 Therapy-responsive microRNAs in plasma samples of 18 head and neck cancer patients

| **miRNA** | **number of patients with ≥ 2 fold up-regulation** | **number of patients with**  **≥ 2 fold down-regulation** | ***p* value** |
| --- | --- | --- | --- |
| miR-590-5p | 1 | 11 | 0.021 |
| miR-574-3p | 1 | 7 | 0.043 |
| miR-425-5p | 2 | 9 | 0.048 |
| miR-885-3p | 0 | 7 | 0.048 |
| miR-21-5p | 3 | 10 | 0.054 |
| miR-106b-5p | 2 | 6 | 0.067 |
| miR-28-3p | 0 | 5 | 0.074 |
| miR-223-5p | 1 | 5 | 0.081 |
| miR-195-5p | 2 | 8 | 0.119 |
| miR-191-5p | 3 | 9 | 0.142 |
| miR-93-5p | 8 | 5 | 0.154 |
| miR-29c-3p | 2 | 7 | 0.181 |
| miR-25-3p | 5 | 3 | 0.196 |
| miR-486-3p | 7 | 3 | 0.196 |
| miR-197-3p | 1 | 5 | 0.212 |
| miR-222-3p | 1 | 7 | 0.212 |
| let-7g-5p | 0 | 3 | 0.304 |
| miR-146b-5p | 3 | 6 | 0.304 |
| let-7b-5p | 2 | 8 | 0.325 |
| miR-628-5p | 1 | 6 | 0.325 |
| miR-146a-5p | 5 | 7 | 0.347 |
| miR-381-3p | 0 | 4 | 0.347 |
| miR-26b-5p | 3 | 6 | 0.370 |
| miR-374b-5p | 3 | 4 | 0.370 |
| miR-140-5p | 3 | 5 | 0.393 |
| miR-885-5p | 3 | 6 | 0.393 |
| miR-26a-5p | 3 | 5 | 0.417 |
| miR-19a-3p | 3 | 5 | 0.442 |
| miR-30c-5p | 4 | 2 | 0.442 |
| miR-454-3p | 3 | 5 | 0.442 |
| miR-186-5p | 4 | 6 | 0.468 |
| miR-483-5p | 1 | 5 | 0.468 |
| miR-92a-3p | 6 | 6 | 0.523 |
| miR-150-5p | 7 | 5 | 0.523 |
| miR-323-3p | 3 | 2 | 0.609 |
| miR-29a-3p | 4 | 3 | 0.671 |
| miR-320a | 5 | 4 | 0.671 |
| miR-451a | 5 | 3 | 0.671 |
| miR-24-3p | 5 | 6 | 0.702 |
| miR-19b-3p | 5 | 4 | 0.766 |
| miR-126-3p | 3 | 5 | 0.766 |
| miR-20b-5p | 4 | 5 | 0.799 |
| let-7e-5p | 5 | 3 | 0.832 |
| miR-17-5p | 4 | 5 | 0.832 |
| miR-30b-5p | 6 | 5 | 0.832 |
| miR-122-5p | 3 | 4 | 0.832 |
| miR-199a-3p | 3 | 4 | 0.832 |
| miR-106a-5p | 3 | 5 | 0.932 |
| miR-486-5p | 5 | 5 | 0.932 |
| miR-16-5p | 5 | 4 | 0.966 |
| miR-20a-5p | 5 | 6 | 0.966 |
| miR-142-3p | 5 | 7 | 0.966 |
| miR-484 | 6 | 5 | 0.966 |
| miR-342-3p | 3 | 4 | 1 |
