# Supplementary material for: Changes in circulating microRNAs after radiochemotherapy in head and neck cancer patients
Source: Radiat Oncol. 2013 Dec 28;8:296. doi: 10.1186/1748-717X-8-296 (PMC3882107; doi:10.1186/1748-717X-8-296)
Supplement: Additional file 3 — Correlation coefficients of normalized Ct values (ΔCt) of plasma miRNAs analyzed with arrays and single assays. [file 1748-717X-8-296-S3.doc]

Additional file 3 Correlation coefficients of normalized Ct values (ΔCt) of plasma miRNAs analyzed with arrays and single assays

|  | **Correlation coefficient (*p* value)** | |
| --- | --- | --- |
| **miRNA** | **ΔCt values prior to treatment** | **ΔCt values post treatment** |
| miR-590-5p | 0.56 (0.029) | 0.85 (0.001) |
| miR-574-3p | 0.75 (0.000) | 0.75 (0.000) |
| miR-425-5p | -0.06 (0.842) | 0.71 (0.003) |
| miR-885-3p | -0.13 (0.652) | 0.00 (--------) |
| miR-21-5p | 0.55 (0.034) | 0.42 (0.122) |
| miR-28-3p | 0.83 (0.000) | 0.85 (0.000) |
| miR-195-5p | 0.49 (0.061) | 0.69 (0.004) |
| miR-191-5p | 0.91 (0.000) | 0.92 (0.000) |
| miR-150-5p | 0.79 (0.001) | 0.65 (0.009) |
| miR-142-3p | 0.63 (0.012) | 0.45 (0.094) |
