# Supplementary material for: Changes in circulating microRNAs after radiochemotherapy in head and neck cancer patients
Source: Radiat Oncol. 2013 Dec 28;8:296. doi: 10.1186/1748-717X-8-296 (PMC3882107; doi:10.1186/1748-717X-8-296)
Supplement: Additional file 4 — Correlation coefficients of normalized Ct values (ΔCt) of miRNAs analyzed with TaqMan single assays in PBMC and plasma. [file 1748-717X-8-296-S4.doc]

Additional file 4 Correlation coefficients of normalized Ct values (ΔCt) of miRNAs analyzed with TaqMan single assays in PBMC and plasma

| **miRNA** | **Correlation coefficient (*p* value)** | |
| --- | --- | --- |
|  | ΔCt values prior to treatment | ΔCt values post treatment |
| miR-574-3p | 0.47 (0.109) | 0.32 (0.294) |
| miR-425-5p | 0.28 (0.350) | 0.00 (0.997) |
| miR-21-5p | 0.24 (0.429) | -0.09 (0.770) |
| miR-28-3p | 0.50 (0.083) | 0.30 (0.317) |
| miR-195-5p | 0.36 (0.231) | -0.05 (0.871) |
| miR-191-5p | 0.23 (0.459) | 0.13 (0.676) |
| miR-150-5p | 0.43 (0.141) | 0.38 (0.206) |
| miR-142-3p | 0.14 (0.652) | 0.11 (0.712) |

PBMC = peripheral blood mononuclear cells
