# Supplementary material for: Changes in circulating microRNAs after radiochemotherapy in head and neck cancer patients
Source: Radiat Oncol. 2013 Dec 28;8:296. doi: 10.1186/1748-717X-8-296 (PMC3882107; doi:10.1186/1748-717X-8-296)
Supplement: Additional file 5 — Significantly deregulated microRNAs in HN1957 primary cell cultures after in vitro radiochemotherapy. [file 1748-717X-8-296-S5.doc]

Additional file 5 Significantly deregulated microRNAs in HN1957 primary cell cultures after *in vitro* radiochemotherapy

| **miRNA** | **fold change** | ***p* value** | **adjusted *p* value** |
| --- | --- | --- | --- |
| miR-181a-3p | 6.71 | 0.002 | 0.018 |
| miR-7-1-3p | 6.09 | 0.002 | 0.018 |
| miR-181a-2-3p | 6.08 | 0.000 | 0.003 |
| miR-454-3p | 3.39 | 0.020 | 0.059 |
| miR-335-5p | 3.18 | 0.019 | 0.059 |
| miR-362-5p | 2.77 | 0.026 | 0.069 |
| miR-29b-1-5p | 1.95 | 0.000 | 0.006 |
| miR-182-5p | 1.83 | 0.015 | 0.055 |
| miR-23a-5p | 1.74 | 0.000 | 0.001 |
| miR-20a-3p | 1.65 | 0.011 | 0.045 |
| miR-4298 | 1.62 | 0.001 | 0.010 |
| miR-224-5p | 1.56 | 0.037 | 0.086 |
| miR-221-5p | 1.55 | 0.004 | 0.026 |
| miR-19b-1-5p | 1.53 | 0.010 | 0.042 |
| miR-148b-3p | 1.46 | 0.012 | 0.045 |
| miR-181a-5p | 1.40 | 0.000 | 0.000 |
| miR-455-5p | 1.38 | 0.005 | 0.027 |
| miR-642b-3p | 1.36 | 0.004 | 0.027 |
| miR-21-3p | 1.34 | 0.000 | 0.009 |
| miR-30a-3p | 1.33 | 0.001 | 0.013 |
| miR-4261 | 1.32 | 0.026 | 0.069 |
| miR-181b-5p | 1.31 | 0.000 | 0.000 |
| miR-23a-3p | 1.27 | 0.005 | 0.027 |
| miR-155-5p | 1.27 | 0.013 | 0.047 |
| miR-425-5p | 1.27 | 0.004 | 0.026 |
| miR-1280_v18.0 | 1.25 | 0.001 | 0.013 |
| miR-98-5p | 1.24 | 0.019 | 0.059 |
| miR-30e-5p | 1.23 | 0.001 | 0.010 |
| miR-1274b_v16.0 | 1.23 | 0.009 | 0.041 |
| miR-7-5p | 1.21 | 0.011 | 0.044 |
| miR-342-3p | 1.21 | 0.008 | 0.039 |
| miR-331-3p | 1.20 | 0.020 | 0.060 |
| miR-151a-5p | 1.20 | 0.009 | 0.041 |
| miR-203a | 1.19 | 0.028 | 0.071 |
| miR-138-5p | 1.18 | 0.027 | 0.071 |
| miR-200a-3p | 1.18 | 0.008 | 0.040 |
| miR-205-3p | 1.17 | 0.000 | 0.006 |
| miR-301a-3p | 1.17 | 0.044 | 0.099 |
| miR-125a-5p | 1.17 | 0.021 | 0.061 |
| let-7f-5p | 1.16 | 0.009 | 0.040 |
| miR-423-5p | 1.15 | 0.001 | 0.011 |
| miR-151a-3p | 1.15 | 0.001 | 0.010 |
| miR-1260a | 1.15 | 0.002 | 0.018 |
| miR-200b-3p | 1.15 | 0.002 | 0.018 |
| miR-4306 | 1.15 | 0.012 | 0.045 |
| miR-503-5p | 1.15 | 0.018 | 0.058 |
| miR-30d-5p | 1.14 | 0.002 | 0.018 |
| miR-31-5p | 1.13 | 0.001 | 0.010 |
| let-7b-5p | 1.13 | 0.020 | 0.059 |
| miR-17-3p | 1.13 | 0.006 | 0.030 |
| miR-31-3p | 1.12 | 0.005 | 0.027 |
| let-7a-5p | 1.11 | 0.021 | 0.061 |
| miR-21-5p | 1.11 | 0.031 | 0.076 |
| miR-1274a_v16.0 | 1.11 | 0.000 | 0.007 |
| miR-320e | 1.11 | 0.001 | 0.011 |
| miR-29b-3p | 1.11 | 0.003 | 0.024 |
| miR-17-5p | 1.09 | 0.019 | 0.059 |
| miR-106b-5p | 0.95 | 0.042 | 0.095 |
| miR-125b-5p | 0.94 | 0.028 | 0.072 |
| miR-92a-3p | 0.92 | 0.022 | 0.061 |
| miR-93-5p | 0.92 | 0.023 | 0.063 |
| miR-141-3p | 0.92 | 0.032 | 0.076 |
| miR-22-3p | 0.91 | 0.012 | 0.045 |
| miR-18a-5p | 0.90 | 0.010 | 0.041 |
| miR-320b | 0.90 | 0.020 | 0.059 |
| miR-24-3p | 0.89 | 0.016 | 0.055 |
| miR-25-3p | 0.88 | 0.007 | 0.037 |
| miR-1260b | 0.87 | 0.001 | 0.010 |
| miR-3911 | 0.84 | 0.014 | 0.050 |
| miR-1914-3p | 0.80 | 0.017 | 0.056 |
| miR-2861 | 0.76 | 0.029 | 0.072 |
| miR-638 | 0.75 | 0.023 | 0.063 |
| miR-3125 | 0.75 | 0.021 | 0.061 |
| miR-940 | 0.75 | 0.008 | 0.040 |
| miR-1305 | 0.74 | 0.018 | 0.058 |
| miR-572 | 0.72 | 0.035 | 0.083 |
| miR-3198 | 0.71 | 0.009 | 0.040 |
| miR-1288 | 0.69 | 0.005 | 0.027 |
| miR-575 | 0.67 | 0.003 | 0.020 |
| miR-324-3p | 0.65 | 0.000 | 0.010 |
| miR-630 | 0.63 | 0.008 | 0.040 |
| miR-4299 | 0.58 | 0.000 | 0.000 |
| miR-1973 | 0.53 | 0.027 | 0.071 |
| miR-1275 | 0.53 | 0.002 | 0.018 |
| miR-1225-5p | 0.52 | 0.004 | 0.025 |
| miR-3663-3p | 0.50 | 0.016 | 0.056 |
| miR-4313 | 0.47 | 0.040 | 0.091 |
| miR-513a-5p | 0.42 | 0.003 | 0.021 |
| miR-494 | 0.41 | 0.010 | 0.042 |
| miR-188-5p | 0.32 | 0.032 | 0.077 |
| miR-3652 | 0.29 | 0.032 | 0.076 |
| miR-513b | 0.19 | 0.002 | 0.016 |
| miR-135a-3p | 0.12 | 0.004 | 0.026 |
