# Supplementary material for: Changes in circulating microRNAs after radiochemotherapy in head and neck cancer patients
Source: Radiat Oncol. 2013 Dec 28;8:296. doi: 10.1186/1748-717X-8-296 (PMC3882107; doi:10.1186/1748-717X-8-296)
Supplement: Additional file 6 — Significantly deregulated microRNAs in HN2092 primary cell cultures after in vitro radiochemotherapy. [file 1748-717X-8-296-S6.doc]

Additional file 6 Significantly deregulated microRNAs in HN2092 primary cell cultures after *in vitro* radiochemotherapy

| **miRNA** | **fold change** | | ***p* value** | **adjusted *p* value** |
| --- | --- | --- | --- | --- |
| miR-193a-5p | 1.66 | 0.000 | | 0.000 |
| miR-7-5p | 1.59 | 0.000 | | 0.000 |
| miR-532-5p | 1.46 | 0.014 | | 0.040 |
| miR-30e-3p | 1.40 | 0.001 | | 0.005 |
| miR-181a-5p | 1.38 | 0.000 | | 0.000 |
| miR-224-3p | 1.35 | 0.003 | | 0.012 |
| miR-181b-5p | 1.31 | 0.000 | | 0.000 |
| miR-4298 | 1.30 | 0.003 | | 0.012 |
| miR-1274b_v16.0 | 1.26 | 0.000 | | 0.000 |
| miR-21-3p | 1.19 | 0.000 | | 0.000 |
| miR-584-5p | 1.19 | 0.013 | | 0.038 |
| miR-17-3p | 1.19 | 0.001 | | 0.003 |
| miR-183-5p | 1.18 | 0.000 | | 0.001 |
| miR-30a-3p | 1.18 | 0.000 | | 0.002 |
| miR-3653 | 1.18 | 0.000 | | 0.002 |
| miR-151a-3p | 1.17 | 0.000 | | 0.000 |
| miR-101-3p | 1.17 | 0.006 | | 0.021 |
| miR-197-3p | 1.17 | 0.008 | | 0.025 |
| miR-452-5p | 1.16 | 0.000 | | 0.000 |
| miR-320e | 1.16 | 0.000 | | 0.000 |
| miR-30e-5p | 1.15 | 0.000 | | 0.000 |
| miR-18b-5p | 1.15 | 0.003 | | 0.012 |
| miR-182-5p | 1.15 | 0.004 | | 0.013 |
| miR-33a-5p | 1.15 | 0.021 | | 0.055 |
| miR-186-5p | 1.15 | 0.035 | | 0.083 |
| miR-30d-5p | 1.15 | 0.000 | | 0.001 |
| miR-320a | 1.14 | 0.000 | | 0.000 |
| miR-454-3p | 1.14 | 0.040 | | 0.093 |
| miR-149-5p | 1.14 | 0.003 | | 0.011 |
| miR-378_v17.0 | 1.14 | 0.000 | | 0.000 |
| miR-335-5p | 1.14 | 0.029 | | 0.071 |
| miR-99b-5p | 1.13 | 0.000 | | 0.000 |
| miR-1274a_v16.0 | 1.13 | 0.000 | | 0.002 |
| miR-320d | 1.12 | 0.002 | | 0.008 |
| miR-4286 | 1.12 | 0.001 | | 0.004 |
| miR-1280_v18.0 | 1.11 | 0.028 | | 0.070 |
| miR-362-5p | 1.11 | 0.022 | | 0.058 |
| miR-128 | 1.11 | 0.036 | | 0.085 |
| miR-222-3p | 1.10 | 0.001 | | 0.006 |
| miR-151a-5p | 1.10 | 0.000 | | 0.000 |
| miR-98-5p | 1.10 | 0.007 | | 0.023 |
| miR-320c | 1.10 | 0.003 | | 0.012 |
| let-7f-5p | 1.09 | 0.041 | | 0.095 |
| miR-20b-5p | 1.09 | 0.029 | | 0.071 |
| miR-1260a | 1.08 | 0.000 | | 0.002 |
| miR-423-5p | 1.08 | 0.011 | | 0.031 |
| miR-15a-5p | 1.08 | 0.009 | | 0.027 |
| miR-148b-3p | 1.08 | 0.011 | | 0.032 |
| miR-17-5p | 1.07 | 0.002 | | 0.008 |
| miR-125a-5p | 1.06 | 0.002 | | 0.009 |
| miR-130b-3p | 1.06 | 0.017 | | 0.046 |
| miR-19a-3p | 1.06 | 0.015 | | 0.041 |
| miR-3651 | 1.06 | 0.005 | | 0.019 |
| miR-455-3p | 1.05 | 0.029 | | 0.071 |
| miR-193b-3p | 1.05 | 0.003 | | 0.012 |
| miR-30a-5p | 1.04 | 0.008 | | 0.025 |
| miR-96-5p | 0.97 | 0.037 | | 0.085 |
| miR-30b-5p | 0.97 | 0.043 | | 0.098 |
| miR-93-5p | 0.96 | 0.001 | | 0.003 |
| let-7i-5p | 0.95 | 0.027 | | 0.070 |
| miR-100-5p | 0.94 | 0.009 | | 0.027 |
| miR-135b-5p | 0.93 | 0.002 | | 0.009 |
| miR-29c-3p | 0.92 | 0.026 | | 0.067 |
| miR-3659 | 0.91 | 0.036 | | 0.085 |
| miR-3656 | 0.90 | 0.009 | | 0.028 |
| miR-1202 | 0.88 | 0.005 | | 0.019 |
| miR-4284 | 0.87 | 0.002 | | 0.008 |
| miR-762 | 0.85 | 0.006 | | 0.022 |
| miR-494 | 0.84 | 0.017 | | 0.046 |
| miR-3665 | 0.83 | 0.000 | | 0.003 |
| miR-3911 | 0.83 | 0.000 | | 0.000 |
| miR-939-5p | 0.81 | 0.000 | | 0.000 |
| miR-4281 | 0.81 | 0.001 | | 0.005 |
| miR-1207-5p | 0.80 | 0.000 | | 0.002 |
| miR-3679-5p | 0.80 | 0.010 | | 0.030 |
| miR-324-3p | 0.79 | 0.002 | | 0.008 |
| miR-940 | 0.79 | 0.002 | | 0.008 |
| miR-1972 | 0.78 | 0.001 | | 0.004 |
| miR-3663-3p | 0.77 | 0.003 | | 0.012 |
| miR-1915-3p | 0.76 | 0.000 | | 0.000 |
| miR-575 | 0.76 | 0.000 | | 0.002 |
| miR-1275 | 0.76 | 0.000 | | 0.000 |
| miR-4299 | 0.75 | 0.000 | | 0.001 |
| miR-638 | 0.73 | 0.000 | | 0.000 |
| miR-1225-5p | 0.73 | 0.000 | | 0.000 |
| miR-630 | 0.72 | 0.000 | | 0.001 |
| miR-513a-5p | 0.68 | 0.001 | | 0.005 |
| miR-2861 | 0.65 | 0.000 | | 0.001 |
| miR-150-3p | 0.64 | 0.023 | | 0.060 |
| miR-572 | 0.63 | 0.000 | | 0.000 |
| miR-16-2-3p | 0.60 | 0.004 | | 0.014 |
| miR-15b-3p | 0.56 | 0.006 | | 0.021 |
| miR-188-5p | 0.50 | 0.000 | | 0.003 |
| miR-513b | 0.33 | 0.000 | | 0.002 |
| miR-1181 | 0.29 | 0.009 | | 0.027 |
